# *α*‐Tocopherol suppresses hepatic steatosis by increasing CPT‐1 expression in a mouse model of diet‐induced nonalcoholic fatty liver disease

**DOI:** 10.1002/osp4.460

**Published:** 2020-10-13

**Authors:** Masanori Tokoro, Koro Gotoh, Yoko Kudo, Yuka Hirashita, Masao Iwao, Mie Arakawa, Mizuki Endo, Junya Oribe, Takayuki Masaki, Koichi Honda, Tetsuya Kakuma, Masataka Seike, Kazunari Murakami, Hirotaka Shibata

**Affiliations:** ^1^ Department of Endocrinology, Metabolism, Rheumatology and Nephrology Faculty of Medicine Oita University Oita Japan; ^2^ Department of Gastroenterology Faculty of Medicine Oita University Oita Japan

**Keywords:** α‐tocopherol, CPT‐1, HepG2 cell, NAFLD model mouse

## Abstract

**Aim:**

Antioxidant therapy for with vitamin E appears to be effective for the treatment of nonalcoholic fatty liver disease　(NAFLD). However, the mechanism of action and optimal therapeutic dosage is unclear. The present study was undertaken to examine whether the effects of α‐tocopherol (α‐Toc) on NAFLD are dose‐dependent in a diet‐induced obese model.

**Methods:**

Male mice were fed standard chow, high‐fat (HF) diet, HF diet with low‐dose, or with high dose of α‐Toc supplementation. Histological findings, triglyceride content, and the levels of protein expression related to fatty acid synthesis/oxidation such as carnitine palmitoyltransferase I (CPT‐1) of liver were evaluated. In addition, 2‐tetradecylglycidic acid (TDGA), a CPT‐1 inhibitor, was administered to mice fed HF diet with low‐dose of α‐Toc. Finally, HepG2 cells in fat‐loaded environment were treated with 0–50 μM α‐Toc.

**Results:**

Treatment of low‐dose of *α*‐Toc decreased HF‐induced hepatic fat accumulation, but this finding was not observed in treatment of high dose of *α*‐Toc. HF‐induced reduction of CPT‐1 was attenuated with low‐dose of *α*‐Toc but not with high dose of *α*‐Toc. TDGA suppressed the improvement of histological findings in liver induced by low‐dose of *α*‐Toc treatment. CPT‐1 expression in HepG2 cells increased in response to low‐dose of *α*‐Toc, but not in high dose.

**Conclusions:**

Dual action of *α*‐Toc on CPT‐1 protein levels was observed. The effect of vitamin E on NAFLD may be not be dose‐dependent.

## 1 INTRODUCTION

Nonalcoholic fatty liver disease (NAFLD) is a clinicopathological diagnosis in which more than 5% of hepatocytes demonstrate macrovesicular steatosis in an individual without a significant history of alcohol intake.^1^ NAFLD encompasses a broad spectrum of metabolic fatty liver disorders ranging from simple steatosis to nonalcoholic steatohepatitis (NASH), which can develop into cirrhosis and liver cancer.^2^, ^3^, ^4^ NASH was originally interpreted with a “dual‐hit” hypothesis, where steatosis (“first hit”), resulting from increased fat accumulation in the liver, predisposes to the initiation of NASH through downstream (“second hit”) pro‐inflammatory mediators.^5^ Previous studies have demonstrated that an imbalance between fatty acid synthesis and oxidation induces fat accumulation in liver, indicating that fatty acid synthesis/oxidation mechanisms are regulated by different hepatic enzymes and transcription factors, which play a central role in controlling lipid homeostasis.^6^, ^7^ Carnitine palmitoyl transferase‐1 (CPT‐1), the mitochondrial gateway for fatty acid entry into the matrix, is a main controller of the hepatic mitochondrial *β*‐oxidation flux.^8^ In the liver, CPT‐1 regulates ∼80% of fatty acid *β*‐oxidation in physiological conditions.^9^ The impairment of *β*‐oxidation in mitochondria induced by the reduction of hepatic CPT‐1 may be a crucial event in the pathogenesis of hepatic steatosis in mice.^10^ In addition, oxidative stress has been implicated as a key factor contributing to hepatic injury in NASH patients. Fat accumulation within hepatocytes enhances mitochondrial reactive oxidative species (ROS) production, which in turn may cause oxidative stress.^11^ The most important cellular damage caused by ROS is peroxidation of membrane lipids, resulting in a generalized alteration of membrane function.^12^

Vitamin E is a potent antioxidant that protects organisms against ROS‐mediated damage.^13^ A previous study has shown that vitamin E can prevent free radicals from entering the cell membrane and helps to maintain the stability of the cell membrane.^14^ In addition, vitamin E inhibits the generation of mitochondrial ROS by increasing the mitochondrial membrane potential and improving mitochondrial function.^15^ Several clinical trials showed that antioxidant therapy with vitamin E was effective in preventing the development of NASH or NAFLD, suggesting that higher intake of vitamin E might be effective in counteracting the increase of oxidative stress found in patients with NASH or NAFLD.^16^, ^17^ On the other hand, a question regarding the safety of a high dose of vitamin E intake has been raised by several meta‐analyses of randomized trials.^18^, ^19^

Taken together, we hypothesized that the therapeutic effect of NAFLD using vitamin E is not dose‐dependent. Here, an in vivo study was conducted to determine the effects of vitamin E on hepatic fat accumulation and the expression of hepatic enzymes and transcription factors related to fatty acid synthesis/oxidation in high‐fat (HF)‐fed mice with different doses of the agent. In addition, in vitro study was performed whether vitamin E treatment also alters the expression of proteins related to hepatic fatty acid synthesis/oxidation in HepG2 cells cultured in fat loaded condition.

## 2 MATERIALS AND METHODS

### 2.1 Animals

Eight‐week‐old male C57BL/6J mice were purchased from KBT Oriental (Fukuoka, Japan). The mice were housed in a light‐, temperature‐, and humidity‐controlled room (lights on/off at 7:00/19:00 h; 21 ± 1°C; 55 ± 5% relative humidity). The mice were allowed free access to chow pellets and water during the experiment. All animals were treated in accordance with the Oita University Guidelines for the Care and Use of Laboratory Animals.

### 2.2 Experimental protocols

Experiment 1: Mice were divided into seven groups (*n* = 10 in each) and given the following diets for 8 weeks: standard diet (control group; 30% protein, 68% carbohydrate, and 12% fat including vitamin E acetate [500 IU/g]; Research Diet); HF diet (HF group; 20% protein, 20% carbohydrate, and 60% fat including vitamin E acetate [500 IU/g]; Research Diet) and HF diet with *α*‐tocopherol (*α*‐Toc) which is one of the natural vitamin E forms supplementation (20, 50, 100, 150, and 200 mg/kg).

Experiment 2: Mice were divided into four groups (*n* = 10 in each) and given the following diets for 8 weeks: (1) standard diet; (2) HF diet; (3) HF diet with 50 mg/kg α‐Toc, one of the natural vitamin E forms, supplementation (low *α*‐Toc group); and (4) HF diet with 200 mg/kg α‐Toc supplementation (high *α*‐Toc group).

Experiment 3: Mice were divided into five groups (*n* = 10 in each) and given the following diets for 8 weeks: (1) standard diet (control group); (2) HF diet (HF group); (3) HF diet after intraperitoneal injection of 2‐tetradecylglycidic acid (TDGA; 30 mg/kg, Sigma, St. Louis, MO, USA), a CPT‐1 inhibitor (HF + TDGA group), (4) HF diet with 50 mg/kg α‐Toc supplementation (HF + low α‐Toc group); and (5) HF diet with 50 mg/dl α‐Toc supplementation after intraperitoneal injection of TDGA (HF + low α‐Toc + TDGA group). In the group that did not receive TDGA, bovine serum albumin (BSA) was given instead of TDGA.

Experiment 4: To determine the change in CPT‐1 expression after *α*‐Toc treatment, HepG2 cells (CosmoBio; 2 × 10^4^) were incubated with 10% fetal calf serum/Roswell park memorial institute (FCS/RPMI)‐1640 (Sigma). After 24 h, the medium was replaced with 1% FCS/RPMI‐1640 and oleic acid (OA) (1 mM). Next, *α*‐Toc (0‐50 µM) was dissolved in ethanol and added at a final concentration of 0–50 μM 24 h after replacement. Then, protein was collected 24 h later and CPT‐1 expression was evaluated by western blotting using CPT‐1a primary antibody (Abcam: ab128568). The ratio of CPT‐1 to *β*‐actin between administrations of α‐Toc was compared.

### 2.3 Serum alanine aminotransferase, triglyceride, alpha‐tocopherol levels, and potential antioxidant assay

At the end of both experiments 1 and 2, the mice were anesthetized with sodium pentobarbital as above and perfused transcardially with isotonic phosphate buffered saline (PBS), followed by 4% paraformaldehyde in 0.1 M PBS. The liver was removed immediately, it was frozen in liquid nitrogen, and then stored at −80°C until protein extraction. Blood samples were withdrawn through cardiac puncture, and the serum was separated and immediately frozen at −80°C until analysis. Serum alanine aminotransferase (ALT) levels and triglyceride (TG) levels were measured using an automatic analyzer (SRL). Serum α‐Toc levels were measured using the following kits in accordance with the method described by the manufacturer: alpha‐tocopherol ELISA Kit (LifeSpan BioSource, Inc). The evaluation of antioxidant power in serum samples was determined by using the “potential antioxidant (PAO)“ test kit (JaICA). This assay evaluated Cu + levels derived by reduction of Cu++ from the action of antioxidant present in the sample. The stable complex between Cu+ and bathocuproine is revealed at 490 nm.

### 2.4 Histological analyses

Liver tissue samples were fixed with 4% paraformaldehyde and embedded in paraffin. Then, 5‐µm‐thick sections were cut, and liver sections were stained with hematoxylin and eosin and examined under a microscope (Olympus). The tissue was analyzed with scoring of the Histological Scoring System for Nonalcoholic Fatty Liver Disease Score and Fibrosis staging by the NASH clinical research network scoring system (NAFLD activity score: NAS). The NAS is defined as the unweighted sum of the scores for steatosis (0–3), lobular inflammation (0–3), and ballooning (0–2).

### 2.5 TG contents in the liver

Liver tissue (100 mg) was homogenized in 2 mL of a solution containing 150 mM NaCl, 0.1% Triton X‐100, and 10 mM Tris using a Polytron homogenizer (NS‐310E; Micro Tech Nichion) for 1 min. The liver TG contents were measured using an automatic analyzer (SRL). A 50‐μL aliquot of the homogenate was removed for the protein assay (Bio‐Rad).

### 2.6 Western blotting

Frozen liver tissue preparations were homogenized with sodium dodecyl sulfate (SDS) sample buffer, clarified by centrifugation, and boiled. The total protein concentration in each tissue sample was quantified by the Bradford method, and 8 µg of total protein per sample was loaded onto 8% SDS‐polyacrylamide gels for electrophoresis. The separated proteins were transferred onto polyvinylidene difluoride membranes (Bio‐Rad Laboratories). The membranes were incubated with primary antibody solution consisting of 5 g/L polyclonal antiserum with specificity for mouse carnitine palmitoyltransferase I (CPT‐1), peroxisome proliferator‐activated receptor alpha (PPARα), uncoupling protein 2 (UCP‐2), acetyl‐CoA carboxylase α (ACACA), sterol regulatory element‐binding protein 1 (SREBP‐1), fatty acid synthase (FAS), and β‐actin. These primary antibodies were purchased from Santa Cruz Biotechnology. Immunoreactive bands were detected by enhanced chemiluminescence (Amersham Life Science) and quantified using National Institutes of Health imaging software. FAS, CPT‐1, PPARα, UCP‐2, ACACA, SREBP‐1, and *β*‐actin of each specimen were measured and determined each protein level as a ratio to that of β‐actin between each group.

### 2.7 Statistics

All data are expressed as the means ± SD. Statistical significance was evaluated using one‐way analysis of variance followed by Tukey's test for post hoc comparisons. For all tests, the level of significance was set at *p* < 0.05. All statistical analyses were performed by using IBM SPSS Statistics version 22.0 for Windows.

## 3 RESULTS

### 3.1 Effect of *α*‐Toc supplementation on serum ALT levels

HF feeding increased serum ALT levels compared with standard feeding (Figure S1). The supplementation of *α*‐Toc at 50 mg/kg for HF diet suppressed the elevation of serum ALT levels induced by HF feeding. Furthermore, the supplementation of *α*‐Toc at 200 mg/kg for HF diet increased serum ALT levels significantly compare to the supplementation of *α*‐Toc at 50 mg/kg. Thus, the supplementations of *α*‐Toc at 50 mg/kg as low‐dose of *α*‐Toc, and *α*‐Toc at 200 mg/kg as high dose of *α*‐Toc were defined.

### 3.2 Low‐dose but not high dose *α*‐Toc improves hepatic damage and lipid accumulation by HF consumption

**FIGURE 1:**
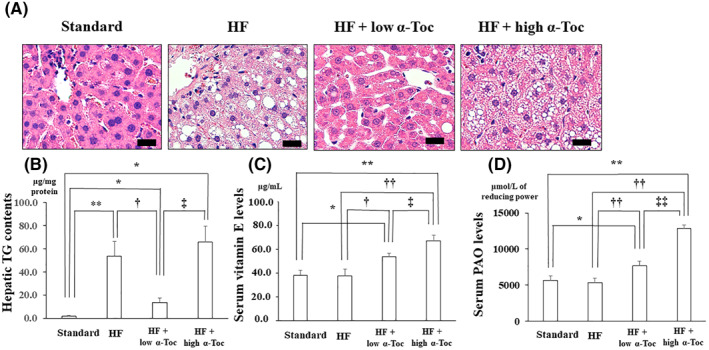
Fat accumulation is decreased with low‐dose but not with high‐dose α‐tocopherol supplementation (A) Representative hematoxylin and eosin staining in the liver derived from each group (B) Quantitation of triglyceride contents in the liver of each group (*n* = 10 in each) (C) Serum α‐tocopherol levels (D) Serum potential antioxidant (PAO) assay. *p < 0.05 versus Standard, **p < 0.01 versus Standard, ^†^ *p* < 0.05 versus high‐fat (HF), ^††^ *p* < 0.01 versus HF, ^‡^ *p* < 0.05 versus HF + low α‐Toc, ^‡‡^ *p* < 0.01 versus HF + low α‐Toc. Standard, standard including vitamin E acetate‐fed; HF, HF including vitamin E acetate‐fed; HF + low α‐Toc, HF‐fed with α‐tocopherol (50 mg/kg) supplementation; HF + high α‐Toc, HF‐fed with α‐tocopherol (200 mg/kg) supplementation. Data are shown as mean ± SD. Scale bar = 20 μm

As shown in Table 1, total body weight, liver weight, and serum ALT levels were increased in HF‐fed mice. The low‐dose but not high dose *α*‐Toc treatment reduced liver weight and serum ALT levels significantly, whereas both doses did not alter total body weight. Serum TG levels were not significantly different among groups. HF‐fed mice developed moderate steatosis with fat accumulation and increased hepatic TG levels in the liver (Figures 1A and 1B).

**TABLE 1:** Body and liver weight, serum ALT, and TG levels with α‐tocopherol supplementation

	Standard	HF	HF + low α‐Toc	HF + high α‐Toc
**Total body weight (g)**	27.6 ± 0.5	43.8 ± 1.3^*^	42.2 ± 1.2^*^	42.2 ± 1.3^*^
**Liver weight (g)**	1.0 ± 0.1	1.4 ± 0.1^*^	1.2 ± 0.1^*,**^	1.3 ± 0.1^*^
**Serum ALT levels (U/L)**	27.1 ± 1.3	84.1 ± 16.2^*^	28.7 ± 1.1^**^	82.1 ± 10.7^*,***^
**Serum TG levels (mg/dL)**	36.7 ± 9.7	32.1 ± 2.9	20.4 ± 1.4	28.5 ± 3.3

*Notes*: Values are the means ± SD. Standard, standard including vitamin E acetate‐fed; HF, HF including vitamin E acetate‐fed; HF + low α‐Toc, HF‐fed with α‐tocopherol (50 mg/kg) supplementation; HF + high α‐Toc, HF‐fed with α‐tocopherol (200 mg/kg) supplementation.

Abbreviations: ALT, alanine aminotransferase; HF, high‐fat; TG, triglyceride.

*p < 0.05 versus Standard, **p < 0.05 versus HF, ***p < 0.05 versus HF + low α‐Toc.

Treatment with a low‐dose of *α*‐Toc decreased these HF‐induced alterations in the liver, however, this improvement was not observed with a high dose of *α*‐Toc treatment (Figures 1A and 1B). Moreover, serum *α*‐Toc levels were significantly increased in high dose of *α*‐Toc more than low‐dose of *α*‐Toc, although both doses elevated serum *α*‐Toc levels compared with Standard as well as HF groups (Figure 1C). Similarly, PAO levels were significantly increased in high dose of *α*‐Toc more than low‐dose of *α*‐Toc, which indicates that high dose of *α*‐Toc has more antioxidant capacity than low‐dose of *α*‐Toc, although both doses elevated PAO levels compared with Standard as well as HF groups (Figure 1D).

In line with hepatic fat accumulation and the hepatic TG content, HF‐induced elevation of the NAS score was also suppressed with the low‐dose but not with the high dose of α‐Toc treatment (Table 2).

**TABLE 2:** Histological findings with α‐tocopherol supplementation

	Standard	HF	HF + low α‐Toc	HF + high α‐Toc
**Steatosis**	0.00 ± 0.00	1.70 ± 0.30^*^	0.40 ± 0.16^**^	1.80 ± 0.36^*^
**Inflammation**	0.00 ± 0.00	0.60 ± 0.27	0.10 ± 0.10	0.50 ± 0.22
**Ballooning**	0.00 ± 0.00	0.50 ± 0.22	0.00 ± 0.00	0.50 ± 0.22
**NAS**	0.00 ± 0.00	2.80 ± 0.77^*^	0.50 ± 0.27^*,**^	2.80 ± 0.77^*,***^

*Notes*: Values are the means ± SD. Standard, standard including vitamin E acetate‐fed; HF, HF including vitamin E acetate‐fed; HF + low α‐Toc, HF‐fed with α‐tocopherol (50 mg/kg) supplementation; HF + high α‐Toc, HF‐fed with α‐tocopherol (200 mg/kg) supplementation.

Abbreviation: HF, high‐fat.

*p < 0.01 versus Standard, ** *p* < 0.05 versus HF, ***p < 0.05 versus HF + low α‐Toc.

### 3.3 A low‐dose of *α*‐Toc enhances the expression of proteins involved in fatty acid oxidation pathways

**FIGURE 2:**
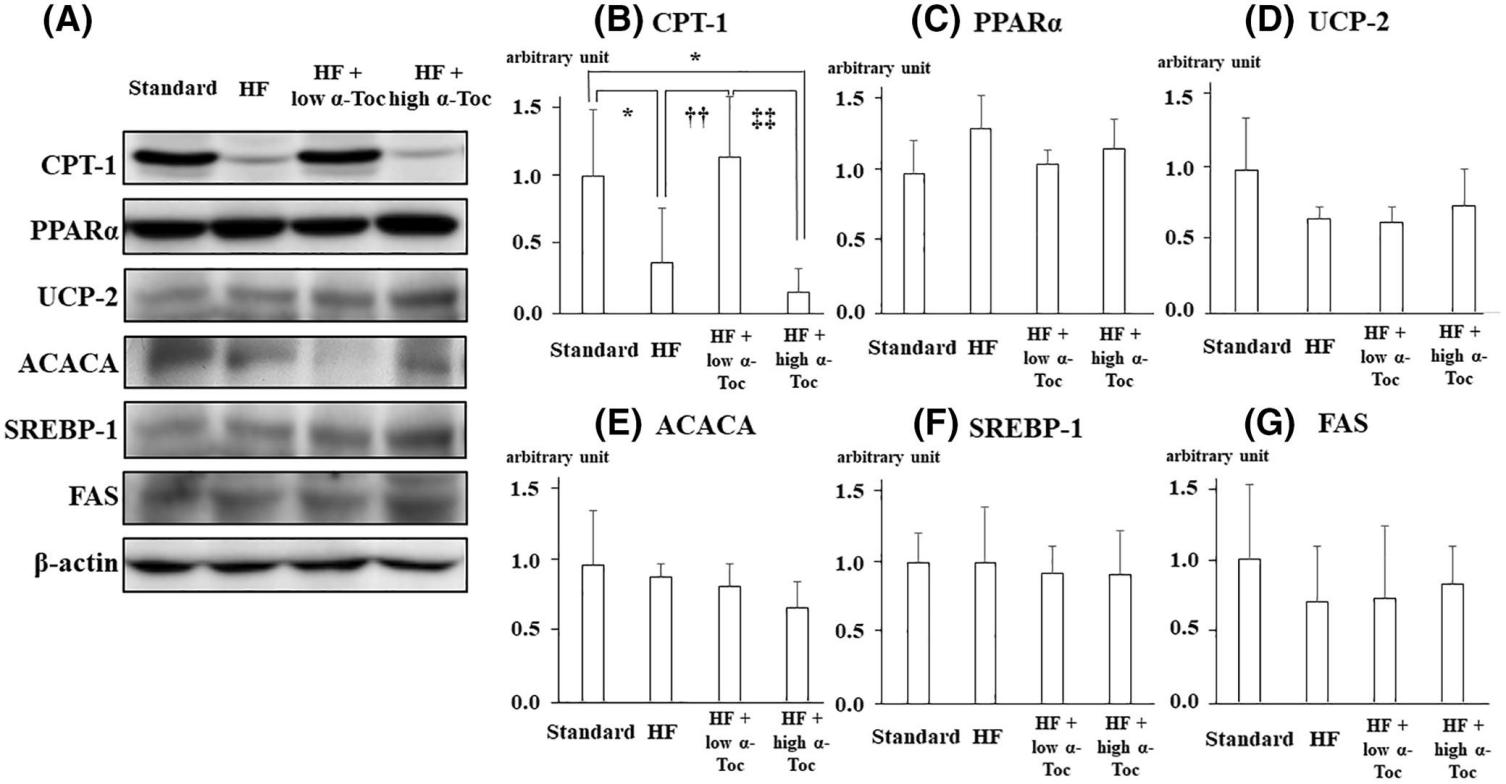
carnitine palmitoyltransferase I (CPT‐1) protein levels in the liver are elevated with low‐dose but not with high‐dose α‐Toc supplementation(A) Western blotting and densitometry analysis were performed to determine hepatic protein levels of mouse CPT‐1, peroxisome proliferator‐activated receptor alpha (PPARα), uncoupling protein 2 (UCP‐2), acetyl‐CoA carboxylase α (ACACA), sterol regulatory element‐binding protein 1 (SREBP‐1), fatty acid synthase (FAS), and β‐actin in each group. (B–G) The relative quantification of CPT‐1 (B), PPARα (C), UCP‐2 (D), ACACA (E), and FAS (G) band intensity divided by β‐actin intensity. Data are shown as mean ± SD. ^*^ *p* < 0.05 versus Standard, ^††^ *p* < 0.01 versus HF, ^‡‡^ *p* < 0.01. Standard, standard including vitamin E acetate‐fed; high‐fat (HF), HF including vitamin E acetate‐fed; HF + low α‐Toc, HF‐fed with α‐tocopherol (50 mg/kg) supplementation; HF + high α‐Toc, HF‐fed with α‐tocopherol (200 mg/kg) supplementation

Protein expression levels related to hepatic fatty acid synthesis/oxidation, such as CPT‐1, PPARα, UCP‐2, ACACA, SREBP‐1, and FAS, in liver tissues were assessed (Figure 2). The levels of CPT‐1 protein expression were significantly decreased in the HF group compared to those of the Standard group. Low‐dose but not high dose α‐Toc treatment increased the levels of CTP‐1 protein (Figures 2A and 2B). However, there were no significant differences in PPARα, UCP‐2, ACACA, SREBP‐1, or FAS expression in the liver among all groups (Figure 2A, Figures 2C–2G). These data indicate that low‐dose *α*‐Toc selectively increased CPT‐1 protein levels in the liver.

### 3.4 TDGA, a specific CPT‐1 inhibitor, suppressed the *α*‐Toc‐induced improvement in hepatic function and fat accumulation in the liver

**FIGURE 3:**
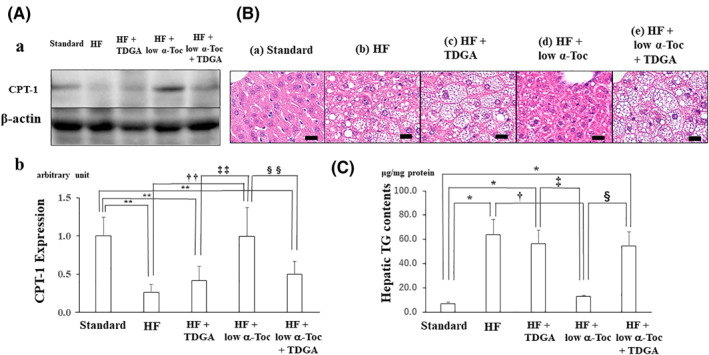
Treatment with a specific carnitine palmitoyltransferase I (CPT‐1) inhibitor attenuates the reduction in fat accumulation in the liver and the improvement in hepatic function induced by low‐dose α‐tocopherol supplementation. (A) 2‐tetradecylglycidic acid (TDGA), a specific CPT‐1 inhibitor, attenuated the expression of CPT‐1 protein and hepatic fat accumulation in the liver. (a) Blots of CPT‐1 and *β*‐actin proteins. (b) The relative quantification of CPT‐1 band intensity divided by *β*‐actin band intensity. (B) Representative hematoxylin and eosin staining in the liver derived from each group. (C) Hepatic triglyceride contents in each group. Data are shown as mean ± SD. ^*^ *p* < 0.05 versus Standard, ^**^ *p* < 0.01 versus Standard, ^†^ *p* < 0.05 versus HF, ^††^ *p* < 0.01 versus HF, ^‡^ *p* < 0.05 versus HF + TDGA, ^‡‡^ *p* < 0.01 versus HF + TDGA, ^§^ *p* < 0.05 versus HF + low α‐Toc. Standard, standard including vitamin E acetate‐fed and bovine serum albumin (BSA) administration; HF, HF including vitamin E acetate‐fed and BSA administration; HF + TDGA, HF‐fed and TDGA (30 mg/kg) administration; HF + low α‐Toc, HF‐fed with α‐tocopherol (50 mg/kg) supplementation and BSA administration; HF + low α‐Toc + TDGA, HF‐fed with α‐tocopherol (50 mg/kg) supplementation and TDGA (30 mg/kg) administration. Scale bar = 20 μm

To further evaluate the effect of CPT‐1 on hepatic accumulation by HF consumption, a specific CPT‐1 inhibitor was used. As shown in Figure 3A, TDGA treatment attenuated the *α*‐Toc‐induced upregulation of CPT‐1 protein levels in the liver. In addition, TDGA also suppressed the *α*‐Toc‐induced improvement in fat accumulation (Figure 3B), reduction in hepatic TG levels (Figure 3C) and NAS (Table 3) in the liver although TDGA alone did not alter HF‐induced hepatic fat accumulation.

**TABLE 3:** Treatment with a specific CPT‐1 inhibitor attenuates the reduction in fat accumulation in the liver and the improvement in hepatic function induced by low‐dose α‐tocopherol supplementation

	Standard	HF	HF + TDGA	HF + low α‐Toc	HF + low α‐Toc + TDGA
**Steatosis**	0.00 ± 0.00	2.5 ± 0.58^*^	2.18 ± 0.75^*^	1.8 ± 0.45^*,**^	2.36 ± 0.50^*,****^
**Inflammation**	0.00 ± 0.00	0.25 ± 0.50	0.18 ± 0.40	0.00 ± 0.00	0.27 ± 0.47
**Ballooning**	0.00 ± 0.00	0.00 ± 0.00	0.18 ± 0.40	0.00 ± 0.00	0.18 ± 0.40
**NAS**	0.00 ± 0.00	2.75 ± 0.50^*^	2.55 ± 1.04	1.8 ± 0.45^*,*** ,**^	2.64 ± 0.50^*,****^

*Notes*: Values are the means ± SD. HF + low α‐Toc. Standard, standard including vitamin E acetate‐fed and BSA administration; HF, HF including vitamin E acetate‐fed and BSA administration; HF + TDGA, HF‐fed and TDGA (30 mg/kg) administration; HF + low α‐Toc, HF‐fed with α‐tocopherol (50 mg/kg) supplementation and BSA administration; HF + low α‐Toc + TDGA, HF‐fed with α‐tocopherol (50 mg/kg) supplementation and TDGA (30 mg/kg) administration.

Abbreviation: BSA, bovine serum albumin; CPT‐1, carnitine palmitoyltransferase I; HF, high‐fat; TDGA, 2‐tetradecylglycidic acid.

*p < 0.01 versus Standard, **p < 0.05 versus HF, ***p < 0.05 versus HF + TDGA, ****p < 0.05 versus HF + low α‐Toc.

### 3.5 Dual mode of action of *α* ‐Toc on CPT‐1 protein levels in HepG2 cells

**FIGURE 4:**
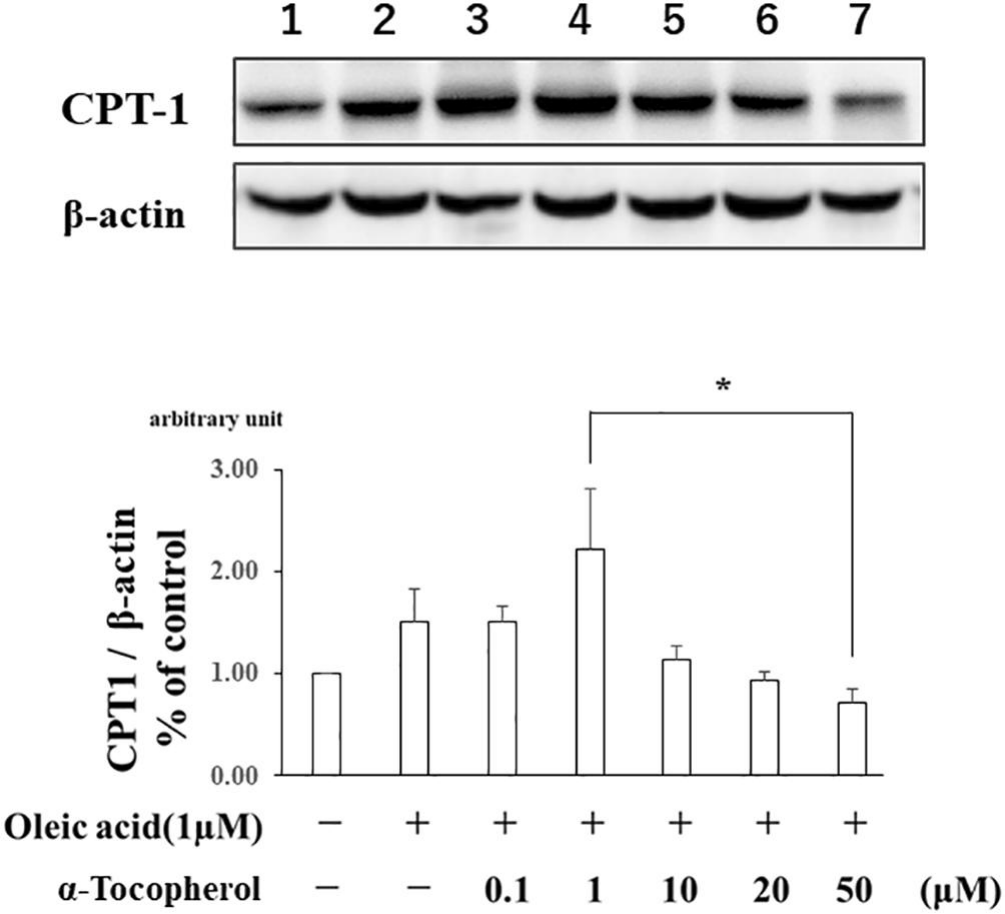
The effect of α‐tocopherol supplementation on the elevation of carnitine palmitoyltransferase I (CPT‐1) protein levels in HepG2 cells has biphasic alteration. Data are shown as mean ± SD. Western blotting and densitometry analysis were performed to determine hepatic protein levels of CPT‐1 and *β*‐actin in each group. ^*^ *p* < 0.05 versus 1 μM α‐tocopherol

Finally, in vitro experiment was performed to determine whether *α*‐Toc‐induced expression levels of CPT‐1 are dose‐dependent, since in vivo experiments showed that a low‐dose of *α*‐Toc alone ameliorated hepatic fat accumulation. OA treatment itself did not promote drastic changes in CPT‐1 expression. However, CPT‐1 protein increased with 0.1 and 1 μM *α*‐Toc. In contrast, *α*‐Toc at greater than 10 μM concentrations decreased the protein to the control level (Figure 4). The in vitro data are actually in line with the in vivo data showing dual modes of action of α‐Toc.

## 4 DISCUSSION

NAFLD is characterized by the fat accumulation in the liver, and such excessive lipid storing consequently worsens normal liver function. C57BL/6 mice were used in this study because it is well‐known that C57BL/6 mice exhibit high sensitivity to obesogenic diets and, therefore, are the most common mouse strain used in experimental NASH.^20^ Several studies have also confirmed hepatic damage through the determination of serum ALT levels in HF‐induced obesity.^21^, ^22^ In accordance with previous findings, this study also observed the elevation of serum ALT levels as well as liver weight, NAS and hepatic TG levels in HF‐fed mice, strongly indicating that HF‐induced fat accumulation in liver is associated with hepatic damage. Interestingly, this study has demonstrated that the supplementation of *α*‐Toc at 50 mg/kg, low‐dose of α‐Toc, improved above alterations and this improvement was disappeared by the supplementation of *α*‐Toc at 200 mg/kg, high dose of α‐Toc, which are compatible with previous reports^23^, ^24^, ^25^ although both serum *α*‐Toc and PAO levels are elevated in high dose of *α*‐Toc more than low‐dose of *α*‐Toc treatment. Considering that low‐dose *α*‐Toc treatment had no effect on the HF‐induced increase in total body weight, a low‐dose of *α*‐Toc might improve HF‐induced liver damage directly.

Hepatic lipogenesis and fatty acid oxidation are crucial pathways that regulate lipid metabolism in the liver. It was examined whether *α*‐Toc treatment influences expressions of endogenous hepatic fatty synthesis proteins such as ACACA, FAS, SREBP‐1, and PPARα, as well as fatty acid oxidation proteins such as CPT‐1 and UCP‐2. This study has demonstrated that HF feeding decreased the expression of CPT‐1, but did not alter other proteins involved in lipid metabolism and compensatory changes in other pathways regulating CPT‐1 expression. Other studies show that short‐term HF diet exposure did not alter the expression of genes involved in hepatic lipogenesis and fatty acid oxidation, such as ACACA, SREBP‐1, FAS, and UCP‐2.^26^, ^27^ Furthermore, the HF‐induced reduction in CPT‐1 was suppressed by the low‐dose of *α*‐Toc treatment but not the high dose of agent. The present study also showed that both low and high doses of *α*‐Toc treatment did not affect other proteins except CPT‐1, thus suggesting that the improvement in HF‐induced liver damage by the low‐dose of *α*‐Toc treatment might be associated with the CPT‐1 pathway, which plays an important role in *β*‐oxidation in hepatocytes. There are eight natural forms of vitamin E: four tocopherols (*α*, *β*, *γ*, and *δ*) and four tocotrienols (*α*, *β*, *γ*, and *δ*). It has been reported that tocotrienol treatment elevates CPT‐1 gene expression using HepG2 cells, indicating that low‐dose vitamin E treatment also alters the expression of CPT‐1 protein at the genetic level.^28^ To further confirm that a low‐dose of *α*‐Toc attenuates the development of NAFLD through the CPT‐1 pathway, an additional experiment exploring the effect of CPT‐1 inhibition was performed. It is known that TDGA, a specific CPT‐1 inhibitor, could induce severe side effects such as hepatic steatosis. The downregulation of CPT‐1 activity caused by TDGA canceled the decrease in serum ALT levels as well as fat accumulation by the low‐dose of *α*‐Toc treatment although TDGA alone did not worse HF‐induced hepatic accumulation and induce hepatic steatosis in this study, strengthening the findings that a low‐dose of α‐Toc improves NAFLD through the regulation of CPT‐1 activity.

CPT‐1, which catalyzes the first step specific to the oxidation of fat, is one of the key enzymes regulating fatty acid metabolism in the mitochondrial membrane.^29^ With increasing CPT‐1 expression, fatty acids could be transported into mitochondria, where they are subject to *β*‐oxidation. It has been observed that CPT‐1 is closely associated with the pathogenesis of NAFLD. Hepatic CPT‐1 expression was significantly suppressed in hepatic injury and fat accumulation, and CPT‐1‐knockdown mice or disorders of carnitine cycle had severe hepatic lipid accumulation and inflammation.^30^, ^31^ The present study also showed that CPT‐1 activity in the liver was increased by low‐dose *α*‐Toc supplementation. These findings suggest a close relationship between the alteration of CPT‐1 expression and development of NAFLD, although it is not possible to conclude that the increase in CPT‐1 expression is responsible for the net result of NAFLD improvement.

*α*‐Toc is the most abundant and potent antioxidant in the eight natural forms of vitamin E, and its effects include scavenging of superoxide radicals. Therefore, higher vitamin E intake might be able to counteract the increase in oxidative stress found in patients with NAFLD. Vitamin E has been studied over the years in the treatment of NAFLD based on this antioxidant property. Studies have provided persuasive evidence that oxidative stress occurs in several different animal models of NAFLD.^32^, ^33^ A previous study demonstrated that CPT‐1 protein is decreased and that CPT‐1 protein oxidation, which might cause a decrease in the amount of active CPT‐1 protein, is promoted in an NAFLD model.^34^ Considering present findings that supplementation with *α*‐Toc, which has an antioxidant effect, elevated CPT‐1 protein as well as improved fat accumulation in the liver, oxidative stress may modify CPT‐1 protein and interfere with fatty acid transport into mitochondria and therefore with their oxidation, leading to an impairment in lipid removal.

Finally, to confirm our suggestion that the effect of *α*‐Toc on CPT‐1 activation is not dose‐dependent, CPT‐1 protein levels were investigated with several different concentrations of *α*‐Toc in an in vitro experiment. HepG2 cells were used because previous other studies have demonstrated that TG accumulation is attenuated with *γ*‐tocopherol reduces TG biosynthesis in HepG2 cells.^28^, ^35^ Interestingly, CPT‐1 protein tended to be increased at between 0.1 and 1 μM *α*‐Toc, and this elevation was not observed at concentrations greater than 10 μM *α*‐Toc, although the OA‐induced reduction in CPT‐1 protein was not observed, indicating that there might be other mechanisms that do not involve the oxidation of CPT‐1 protein induced by oxidative stress. Other studies confirmed that the hepatic TG‐lowering effect of vitamin E could be partly explained by increasing CPT‐1 gene expression in HepG2 cells, which would be compatible with present findings.^36^, ^37^ Considering these results, there is the possibility that the dual action of *α*‐Toc on CPT‐1 protein levels in HepG2 cells observed in the present study might be associated with the degree of oxidative stress. As shown in Figures 1C and 1D, *α*‐Toc supplementation increases serum *α*‐Toc levels and has antioxidant effects in a dose‐dependent manner. Therefore, the biphasic effects of α‐Toc were hypothesized on hepatic fat accumulation as follows. The low‐dose *α*‐Toc ameliorates hepatic fat accumulation due to elevation of CPT‐1 levels whereas high dose *α*‐Toc reciprocally worsens hepatic changes due probably to toxic pharmacological effects of *α*‐Toc as some papers have already reported.^38^, ^39^

A recent meta‐analysis showed improvements in body mass index, ballooning, fibrosis and histology, but such improvements were not much significant to draw a firm conclusion.^40^ The present study suggests the clinically useful effect of vitamin E on NAFLD might not be dose‐dependent. To the best of our knowledge, this is the first study to evaluate whether vitamin E supplementation suppresses the development of NAFLD by elevating CPT‐1 protein levels in the liver although the details are uncertain.

This study is limited by the use of an animal model that cannot fully replicate the pathology of human NAFLD. The reduction in CPT‐1 activity observed in this NAFLD model may not be easily replicated in humans. Further studies examining the dosing of vitamin E in humans are still required. While this study has suggested a potential mechanistic pathway involved in the efficacy of *α*‐Toc supplementation, the mechanisms responsible for the beneficial effect of vitamin E therapy were not fully elucidated because there are many factors, such as lipotoxicity and inflammatory cytokines, in addition to oxidative stress, in the progression to NAFLD. Plus, several studies have shown that daily administration of vitamin E increases the risk of developing prostate cancer in healthy men and that high‐dose vitamin E supplementation increases mortality.^41^, ^42^ The timing and the duration of the treatment with vitamin E are important factors. These are often determined optionally and it is difficult to be determined adequately.^43^ Therefore, the effectiveness and safety of using vitamin E as a form of treatment require further study.

In conclusion, we demonstrated that a low‐dose of *α*‐Toc protects against NAFLD‐induced liver injury through an increase in CPT‐1 protein, which regulates mitochondrial lipid metabolism in the liver, whereas a high dose of *α*‐Toc suppresses these alterations. Therefore, the useful effect of vitamin E on NAFLD might not be dose‐dependent. In the future, large multicenter, double‐blinded randomized controlled trials to investigate the appropriate dose of vitamin E administration on NAFLD are warranted**.**

## CONFLICT OF INTEREST

The authors declare that they have no conflicts of interest. Informed consent was obtained from all individual participants included in the study.

## Supporting information

Supplementary Material
