# Supplementary figures and images for: α‐Tocopherol suppresses hepatic steatosis by increasing CPT‐1 expression in a mouse model of diet‐induced nonalcoholic fatty liver disease
Source: Obes Sci Pract. 2020 Oct 13;7(1):91–9. doi: 10.1002/osp4.460 (PMC7909598; doi:10.1002/osp4.460)

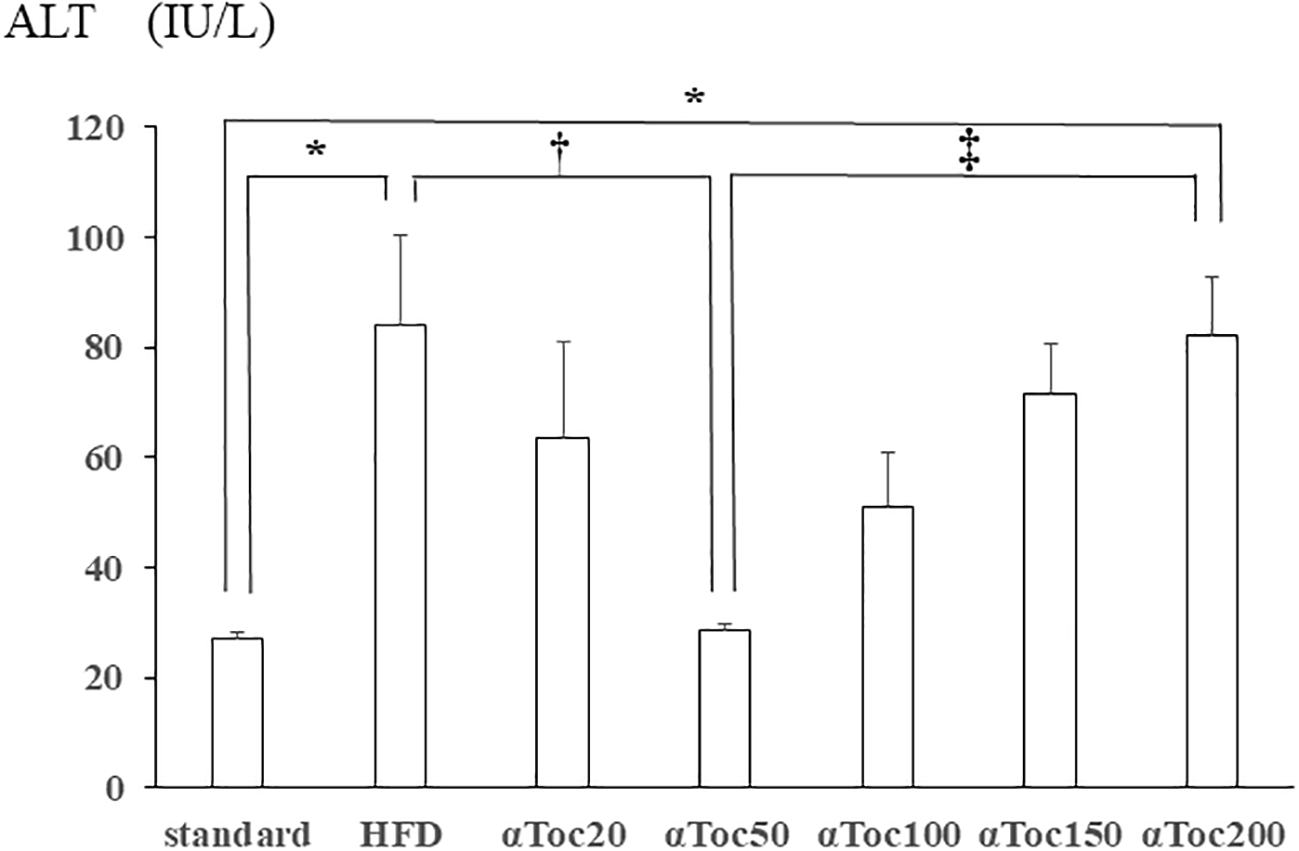

Supplement: Supplementary file 1 — Supplementary Material [file OSP4-7-91-s001.tif]
